# Homocysteine, HHcy, H-type hypertension and dizziness: an NHANES analysis

**DOI:** 10.3389/fneur.2025.1550568

**Published:** 2025-07-14

**Authors:** Yiyin Liang, Tianjie Lai, Juan Feng

**Affiliations:** ^1^Department of Otorhinolaryngology, The First Affiliated Hospital of Xinjiang Medical University, Urumqi, China; ^2^Department of Spine Surgery, The Affiliated Yuebei People's Hospital of Shantou University Medical College, Shaoguan, China

**Keywords:** dizziness, homocysteine, hyperhomocysteinemia (HHcy), H-type hypertension, balance problems, positional dizziness, falling problems

## Abstract

**Background:**

Homocysteine (Hcy) is associated with various diseases, but its specific relationship with different types of dizziness remains unclear.

**Objectives:**

This study utilizes NHANES cross-sectional data to investigate the associations between Hcy levels, H-type hypertension, and various symptomatic dizziness, aiming to provide new insights for clinical diagnosis and treatment.

**Materials and methods:**

This cross-sectional study analyzed 6,970 participants from NHANES (1999–2004) using weighted logistic regression, trend tests, restricted cubic spline analysis, and subgroup analysis.

**Results:**

Elevated Hcy levels and H-type hypertension showed significant positive associations with various symptomatic dizziness. HHcy showed the strongest association with fall risk (OR = 1.83, 95% CI: 1.24–2.77), while H-type hypertension was most strongly associated with any symptomatic dizziness (OR = 1.75, 95% CI: 1.34–2.28). No significant associations were found with positional dizziness. Trend analysis indicated a significant upward trend in the risk of any symptomatic dizziness, balance problems, and falling problems. RCS analysis demonstrated non-linear relationships between Hcy levels and various symptomatic dizziness, including any symptomatic dizziness, balance problems, and falling problems.

**Conclusions and significance:**

This study revealed that Hcy levels, HHcy, and H-type hypertension were significantly associated with various symptomatic dizziness. Recognizing and controlling HHcy and H-type hypertension are vital for dizziness management and diagnosis.

## Introduction

Dizziness, a common clinical syndrome characterized by disturbed spatial orientation and balance, affects 15–36% of the US population (1). Originally, Drachman and Hart (2) classified it into four categories: vertigo, presyncope, disequilibrium, and light-headedness, building upon this foundation, the Bárány Society’s International Classification of Vestibular Disorders (ICVD) has proposed a symptom-driven approach to vestibular disorders (3–5). This framework recognizes that dizziness can arise from a wide range of etiologies beyond the vestibular system, including cardiovascular (6, 7), neurological (8), metabolic (9), psychological factors (10) and musculoskeletal factors (11). These multifactorial origins underscore the complexity of dizziness and the challenges in its accurate diagnosis and effective management.

Clinical management of dizziness presents substantial challenges due to its non-specific and heterogeneous symptom presentation, along with frequently inconclusive objective examinations (12, 13). Diagnostic complexities delay accurate diagnosis while leading to excessive imaging studies to exclude severe conditions, thereby exerting multifaceted impacts on both patients and healthcare systems (14, 15). Such a diagnostic and treatment pattern subsequently increases patients’ healthcare burden, significantly degrades quality of life, and elevates their risks of falls, hospitalization, and disability (16–18). Estimates suggest that dizziness-related disorders, particularly vertigo, pose a significant economic challenge to the U.S. healthcare system, with annual direct costs approaching $50 billion (19, 20). When indirect costs, such as productivity losses, are included, this financial burden is further magnified (21). As the population ages, the incidence of dizziness continues to rise, placing increasing strain on global healthcare systems (22, 23). This underscores the urgent need to explore risk factors for dizziness from a symptomatic perspective, providing strategies for diagnosis and treatment.

Homocysteine (Hcy) is present in all body cells, and its abnormal metabolism has been associated with a variety of diseases, including cardiovascular disease (24), neurodegenerative diseases (25), and osteoporosis (26). However, the possible association between vertigo and Hcy levels has garnered significant attention from researchers in recent years (27). Some small-scale studies have suggested a potential link between HHcy (H-type hypertension) and certain types of dizziness, but these findings have not yet been validated in large-scale population studies (28). In particular, considering that hypertension itself may be associated with certain types of dizziness, the association of the coexistence of hypertension and HHcy with dizziness becomes a question worth exploring. However, there is still a lack of systematic studies on the association between Hcy, HHcy, and H-type hypertension and different types of dizziness, such as any symptomatic dizziness, balance problems, falling problems, and positional dizziness. This study utilizes NHANES data to investigate these relationships, aiming to provide evidence for the associations between Hcy, HHcy, H-type hypertension, and dizziness. Furthermore, these findings may contribute to the optimization of diagnostic, preventive, and therapeutic strategies for dizziness.

## Materials and methods

### Data source and study population

**Figure 1:**
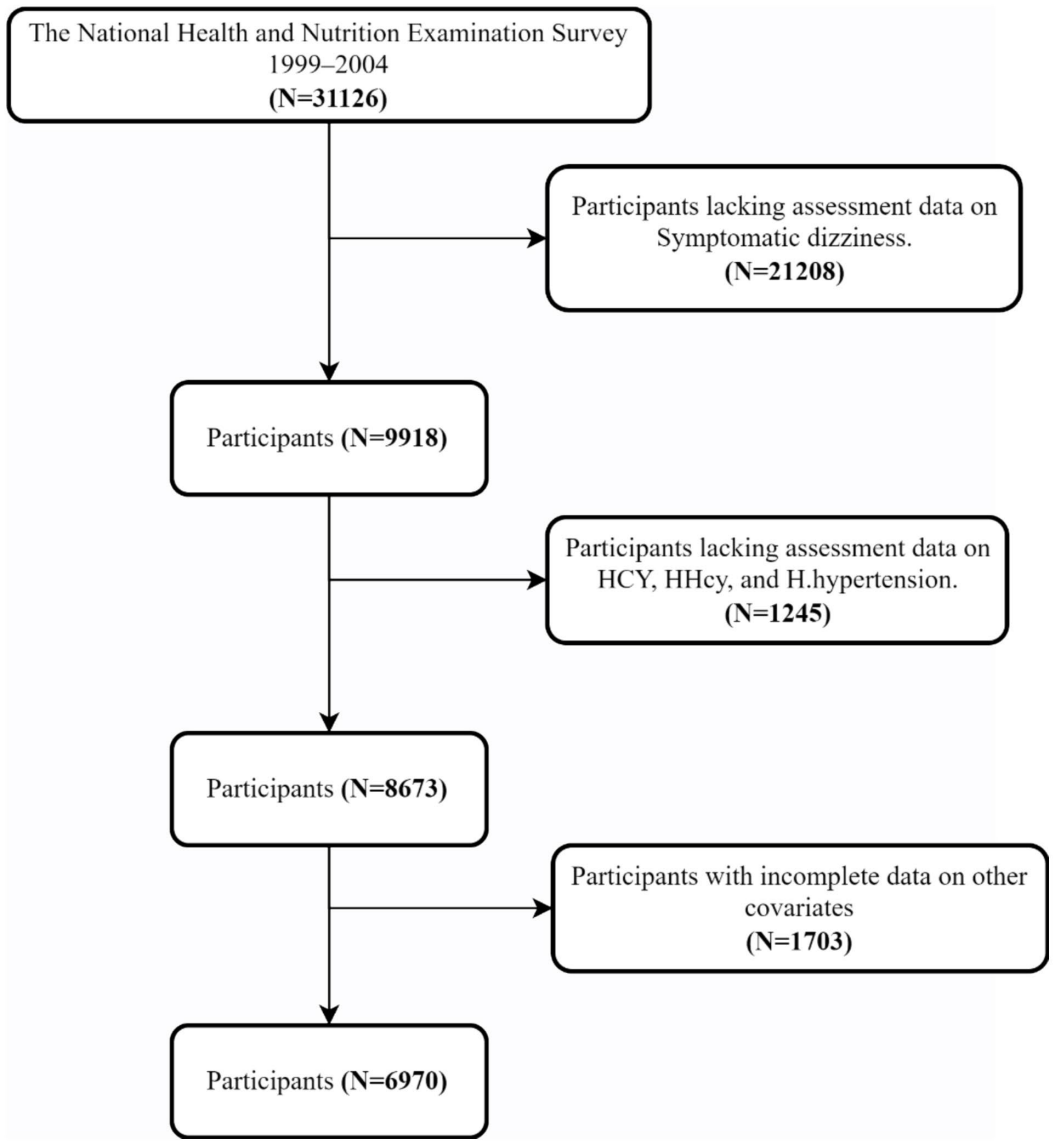
Participant selection flowchart.

NHANES is a nationally representative health survey conducted by the National Center for Health Statistics (NCHS). The survey uses a complex multistage sampling design to assess the health and nutritional status of the non-institutionalized population in the United States. Since 1999, NHANES has released data every 2 years and implemented strict quality assurance measures, including regular reviewer reliability assessments, to ensure data accuracy and continuous methodological improvements. This study analyzed NHANES data from 1999 to 2004, with the specific study period determined by the availability of key variables: Hcy measurements were available from 1999 to 2006, while symptomatic dizziness data were available from 1999 to 2004. Although the data are not recent, this timeframe ensured both variable completeness and a sufficiently large sample size for robust statistical analysis. The findings remain relevant for understanding the baseline relationship between Hcy and dizziness. We excluded participants with missing data on evaluating symptomatic dizziness, Hcy, HHcy, and H-type Hypertension, as well as participants with missing other key covariates. A total of 6,970 participants were finally included in the analysis (Figure 1 for details). The NCHS Ethics Review Board approved the study protocol,^1^ and all participants provided informed consent.

### Hcy and HHcy assessment

The quantification of serum Hcy levels was performed utilizing Abbott Diagnostics’ fully automated Fluorescence Polarization Immunoassay (FPIA) methodology. The method used the IMx system from 1999 to 2001 and was switched to the Axsym system from 2002 to 2006. The NHANES study showed that the long-term coefficient of variation of Hcy concentrations during these two periods remained at 3–5%, reflecting the method’s stability. Cross-validation studies of the two systems confirmed the comparability of the data. The FPIA method is suitable for large-scale epidemiological studies and provides a reliable basis for accurately determining Hcy concentrations (29, 30). While the definition of hyperhomocysteinemia (HHcy) varies, this study adopted the widely accepted clinical and research threshold of >15 μmol/L for plasma Hcy levels (31–34). To ensure the robustness of our findings, we also conducted a sensitivity analysis using the alternative threshold of >10 μmol/L.

### Dizzy assessment

Symptoms of dizziness were assessed using the following questions from the NHANES database: “In the past 12 months, have you experienced dizziness, difficulty with balance, or problems with falls?” A positive response was defined as any symptomatic dizziness. Respondents who answered yes to this question were further asked to determine three dizziness variables: dizziness problems, balance problems, or problems with falls. Positional dizziness was defined based on a positive response to the question “Do you feel dizzy when you turn over in bed?.” Due to questionnaire design limitations, this study could not further distinguish between specific types of dizziness, such as rotational vertigo, lightheadedness, or feelings of disequilibrium. While this question does not differentiate between specific types of vestibular symptoms as defined by the ICVD, it provides a broad assessment of balance-related issues that participants found bothersome. In this study, we focused on symptomatic dizziness, characterized by participants’ self-reported experiences of dizziness, vertigo, falling, or balance problems, rather than relying on modified Romberg test indicators. This approach differs from the NHANES method, which employs mCTSIB as a screening tool for vestibular balance function (35, 36). Our strategy has been supported by previous large-scale clinical studies (37).

### Definitions of covariates

To comprehensively evaluate potential influencing factors, this study included multiple covariates to analyze the main variables. These covariates included demographic characteristics (age, sex, race), lifestyle factors (alcohol use, diet quality, total energy intake, physical activity, smoking status), and socioeconomic status (income level, marital status, education attainment). Race was categorized as non-Hispanic Black, non-Hispanic White, and Other/Multi-racial. Diet quality was assessed using the Healthy Eating Index (HEI, 2015 version). Physical activity was quantified using the Metabolic Equivalent of Task (MET). Smoking status was classified as never, former, or current smoker. The income level was divided into poverty and non-poverty. Education attainment was categorized as high school or below, college graduate or above.

### Statistical analysis

This study incorporated NHANES’s complex sampling design, using sample weights, stratification, and clustering in all analyses. Baseline characteristics were compared by symptomatic dizziness status. Continuous variables (means, 95% CIs) were analyzed using the Wilcoxon rank-sum test for complex survey samples, while categorical variables (percentages, 95% CIs) were analyzed using the chi-square test with Rao & Scott’s for second-order correction (38). Weighted logistic regression assessed associations between Hcy, HHcy, H-type hypertension, and symptomatic dizziness across progressive models: Model 1 (unadjusted), Model 2 (adjusted for age, sex, race), and Model 3 (fully adjusted). Trend tests evaluated dose–response relationships between Hcy quartiles and dizziness outcomes, with the lowest quartile (Q1) serving as reference. Restricted cubic splines (RCS), with knots selected via Akaike Information Criterion (AIC) and adjusted for confounders, explored potential nonlinear Hcy-dizziness associations. Subgroup analyses assessed robustness and interactions. Analyses were performed in R (version 4.3.1), with two-tailed *p* < 0.05 indicating significance.

## Results

### Characteristics of participants

A total of 6,970 participants were enrolled in this study and grouped based on their dizziness condition. The weighted characteristics of the participants are detailed in Table 1. The analysis revealed that the average age of individuals without any dizziness symptoms was 55 years, while the average age of those with dizziness symptoms was 60 years. The group with dizziness symptoms tended to be predominantly female and had a relatively lower income level and a lower level of education. Additionally, this group was more likely to abstain from alcohol, tended to smoke, and exhibited a higher prevalence of conditions such as stroke, diabetes, and cardiovascular diseases.

**Table 1:** Characteristics of participants.

Characteristic	Any symptomatic dizziness (*N* = 6,970)		*p* value^c^
	No, *N* = 5177^a^^,^^b^	Yes, *N* = 1793^a^^,^^b^	
Age, years	55 [55, 56]	60 [59, 60]	**<0.001**
Sex, %			**<0.001**
Male	51 [49, 53]	38 [36, 41]	
Female	49 [47, 51]	62 [59, 64]	
Race, %			**0.042**
Other/multiracial	12 [9.8, 16]	16 [11, 22]	
Non-Hispanic black	8.5 [6.9, 10]	8.8 [6.7, 11]	
Non-Hispanic white	79 [76, 82]	75 [70, 80]	
Income level, %			**<0.001**
Poor	7.8 [6.7, 9.1]	19 [15, 22]	
Not poor	92 [91, 93]	81 [78, 85]	
Education attainment, %			**<0.001**
High school or below	43 [40, 46]	58 [53, 62]	
College graduate or above	57 [54, 60]	42 [38, 47]	
Alcohol use, %			**<0.001**
Non drinker	11 [9.2, 14]	17 [14, 20]	
Drinker	89 [86, 91]	83 [80, 86]	
Smoke status, %			**0.020**
Never	48 [46, 50]	43 [39, 48]	
Former	33 [31, 35]	34 [31, 37]	
Now	19 [18, 21]	23 [20, 27]	
BMI, kg/m^2^	28.6 [28, 29]	28.8 [28, 29]	0.6
Stroke, %	1.9 [1.6, 2.4]	8.4 [7.0, 10]	**<0.001**
Heart disease, %	10 [9.3, 12]	24 [21, 26]	**<0.001**
Diabetes, %	8.3 [7.5, 9.1]	17 [15, 20]	**<0.001**

a Mean; %.

b CI = Confidence Interval.

c Wilcoxon rank-sum test for complex survey samples; chi-squared test with Rao & Scott’s second-order correction.A *p* value of < 0.05 was regarded as statistically significant in bold.

### Association between Hcy, HHcy, and H-type hypertension and different symptomatic dizziness

The research findings indicate that levels of Hcy, HHcy, and H-type hypertension are significantly positively associated with symptomatic dizziness. In the unadjusted model (Model 1) and the model adjusted for core demographic factors (Model 2), these associations are significant for most types of symptomatic dizziness (as detailed in Table 2). However, in Model 3 with full covariate adjustment, the associations between Hcy and various types of symptomatic dizziness weaken, with most losing statistical significance. Despite this attenuation, the associations between HHcy and H-type hypertension with symptomatic dizziness remain significant after full adjustment. Notably, HHcy shows the strongest association with the risk of falling problems (OR = 1.83, 95% CI: 1.24–2.77), while H-type hypertension is most strongly associated with any symptomatic dizziness (OR = 1.75, 95% CI: 1.34–2.28). Importantly, no significant association was found between Hcy, HHcy, or H-type hypertension and positional dizziness in any of the models. This suggests that positional dizziness may have different pathological mechanisms or risk factors.

**Table 2:** Association between Hcy, HHcy, and H-type hypertension and different symptomatic dizziness.

Model^a^	Characteristic	Any symptomatic dizziness			Dizziness problems			Balance problems			Falling problems			Positional dizziness		
		OR^b^	95% CI^b^	*p*-value	OR^b^	95% CI^b^	*p*-value	OR^b^	95% CI^b^	*p*-value	OR^b^	95% CI^b^	*p*-value	OR^b^	95% CI^b^	*p*-value
Model 1	HCY	1.04	1.02, 1.07	**0.001**	1.03	1.01, 1.05	**0.005**	1.05	1.02, 1.08	**<0.001**	1.07	1.03, 1.10	**<0.001**	1.01	0.98, 1.05	0.4
	HHcy															
	No	—	—		—	—		—	—		—	—		—	—	
	Yes	2.33	1.87, 2.91	**<0.001**	1.94	1.49, 2.53	**<0.001**	2.33	1.84, 2.95	**<0.001**	3.45	2.44, 4.88	**<0.001**	1.35	0.81, 2.25	0.2
	H-type hypertension															
	No	—	—		—	—		—	—		—	—		—	—	
	Yes	2.75	2.18, 3.47	**<0.001**	2.23	1.61, 3.10	**<0.001**	2.71	1.99, 3.69	**<0.001**	3.55	2.44, 5.16	**<0.001**	0.87	0.42, 1.79	0.7
Model 2	Hcy	1.04	1.01, 1.07	**0.018**	1.03	1.00, 1.05	**0.025**	1.04	1.01, 1.07	**0.012**	1.05	1.01, 1.09	**0.016**	1.02	0.99, 1.04	0.2
	HHcy															
	No	—	—		—	—		—	—		—	—		—	—	
	Yes	1.91	1.48, 2.46	**<0.001**	1.76	1.33, 2.32	**<0.001**	1.74	1.34, 2.26	**<0.001**	2.38	1.64, 3.46	**<0.001**	1.28	0.77, 2.11	0.3
	H-type hypertension															
	No	—	—		—	—		—	—		—	—		—	—	
	Yes	2.14	1.67, 2.74	**<0.001**	1.96	1.39, 2.74	**<0.001**	1.91	1.38, 2.65	**<0.001**	2.27	1.55, 3.31	**<0.001**	0.77	0.38, 1.56	0.5
Model 3	Hcy	1.02	1.00, 1.05	0.094	1.02	1.00, 1.04	0.10	1.03	1.00, 1.05	0.052	1.04	1.00, 1.08	0.062	1.01	0.97, 1.05	0.7
	HHcy															
	No	—	—		—	—		—	—		—	—		—	—	
	Yes	1.60	1.21, 2.10	**0.002**	1.47	1.12, 1.93	**0.007**	1.44	1.11, 1.87	**0.008**	1.83	1.24, 2.70	**0.003**	1.01	0.59, 1.72	>0.9
	H-type hypertension															
	No	—	—		—	—		—	—		—	—		—	—	
	Yes	1.75	1.34, 2.28	**<0.001**	1.62	1.15, 2.27	**0.007**	1.52	1.09, 2.12	**0.015**	1.63	1.11, 2.38	**0.014**	0.57	0.26, 1.27	0.2

Model 1: Not adjusted. Model 2: Adjusted Age, Sex, Race. Model 3: Adjusted Age, Sex, Race, Income level, Education attainment, Alcohol use, Smoke status, BMI, Stroke, Heart disease, Diabetes.

a Models: Not adjusted; Model 2: Adjusted Age, Sex, Race; Model 3: Adjusted Age, Sex, Race, Income level, Education attainment, Alcohol use, Smoke status, BMI, Stroke, Heart disease, Diabetes.

b OR, Odds Ratio, CI, Confidence Interval.A *p* value of < 0.05 was regarded as statistically significant in bold.

### Trend tests of association between Hcy and different symptomatic dizziness

**Figure 2:**
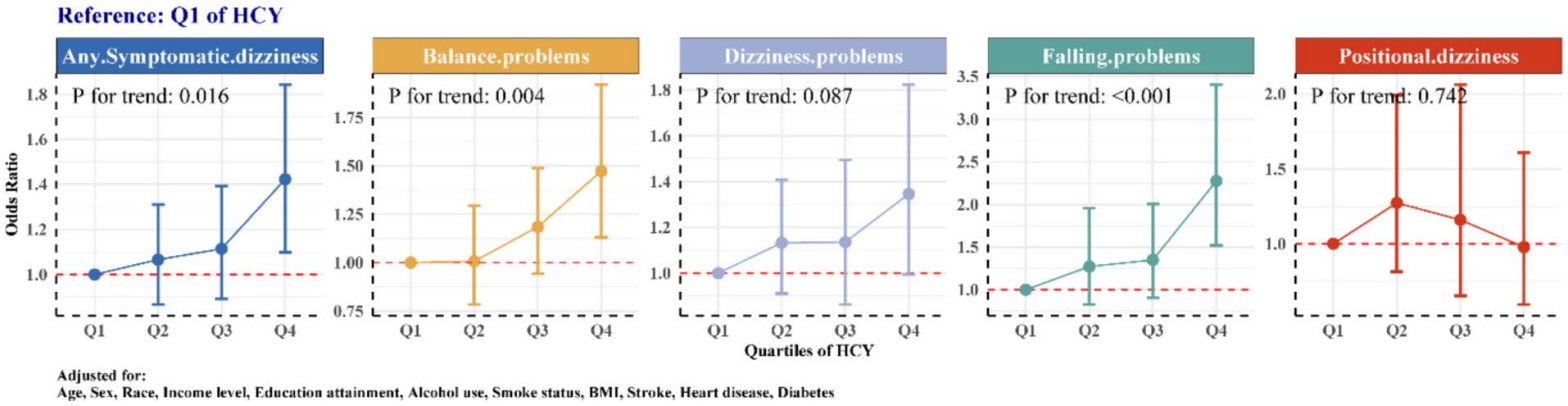
Trend tests of association between Hcy and different symptomatic dizziness.

We categorized the levels of Hcy into quartiles and conducted a trend analysis (detailed in Figure 2). The results indicate that using the lowest Hcy level group (Q1) as a reference, there is a generally increasing trend in the risk of symptomatic dizziness, such as any symptomatic dizziness, balance problems, and falling problems, as Hcy levels rise. This trend is statistically significant (p for trend < 0.0001). However, no such trend was observed in individuals experiencing dizziness problems or positional dizziness.

### Restricted cubic spline (RCS) analysis of the association between Hcy and different symptomatic dizziness

**Figure 3:**
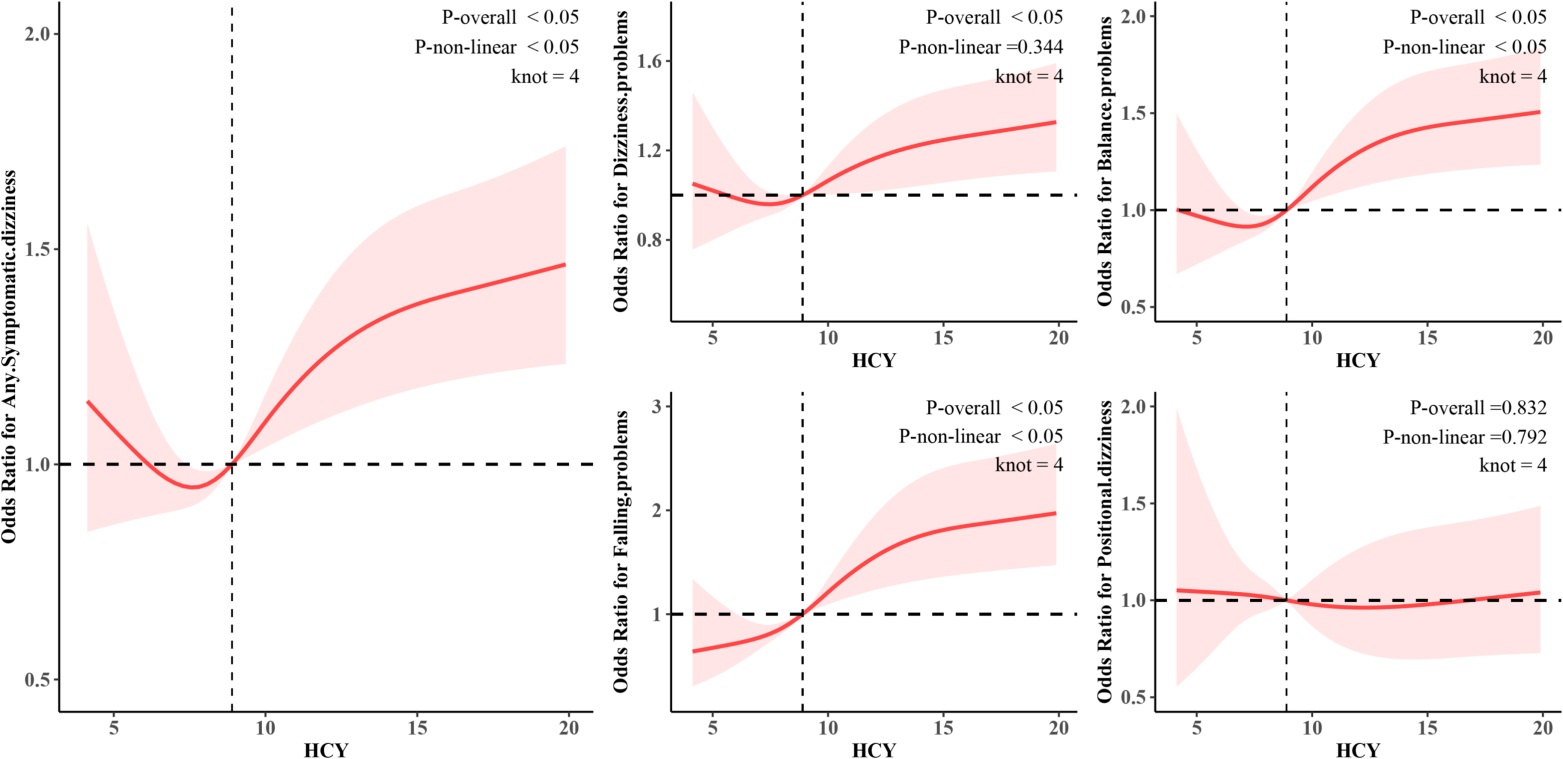
Restricted cubic spline (RCS) analysis of the association between Hcy and different symptomatic dizziness.

The results of the weighted RCS analysis (Figure 3 for details) indicate a significant nonlinear relationship between Hcy and any symptomatic dizziness, balance problems, and falling problems (p for nonlinear < 0.05). Specifically, Hcy exhibits a “J”-shaped relationship with any symptomatic dizziness and balance problems, while it shows an “S”-shaped relationship with falling problems. For any symptomatic dizziness and balance problems, a slight increase in low Hcy levels is associated with a minor decrease in risk; however, once Hcy levels exceed a certain threshold, the risk begins to rise significantly. The “S”-shaped association with falling problems suggests both a threshold effect and a saturation effect: after Hcy levels surpass a critical value, the risk increases significantly, but the rate of increase slows down at higher levels. It is noteworthy that Hcy showed a predominantly linear association with dizziness problems (p-overall < 0.05, p-nonlinear = 0.344), whereas no significant association was found with positional dizziness (p-overall = 0.832, p-nonlinear = 0.792).

### Subgroup analysis of the relationship between Hcy, HHcy, and H-type hypertension and different symptomatic dizziness

**Figure 4:**
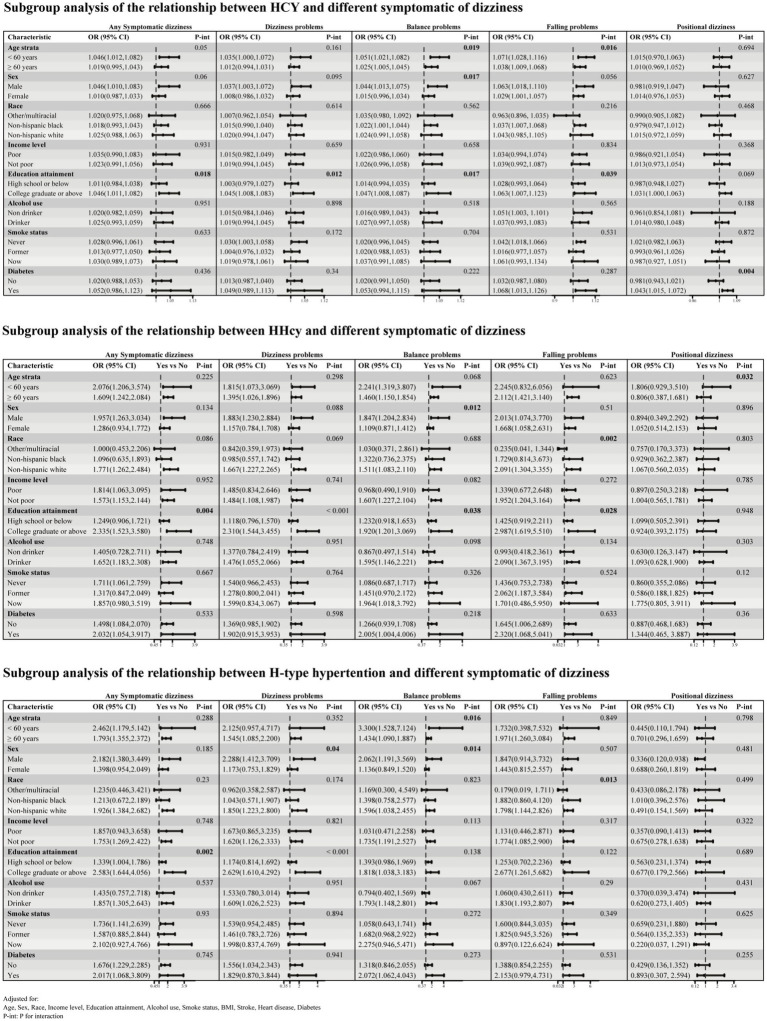
Subgroup analysis of the relationship between Hcy, HHcy, and H-type hypertension and different symptomatic dizziness.

The subgroup analysis (Figure 4) revealed that the associations between Hcy, HHcy, and H-type hypertension with dizziness symptoms are influenced by multiple factors. Gender and education level exhibited the most significant interactions, with gender playing a crucial moderating role in these relationships. In men, the positive correlation between Hcy and HHcy with balance problems is more pronounced, and the association of H-type hypertension with dizziness and balance problems is also more prominent in this group. Education level also showed extensive interaction effects, but the influencing factors differed: for Hcy and HHcy, education level interacted with different types of symptomatic dizziness (including any dizziness symptoms, dizziness problems, balance problems, and falling problems). In contrast, for H-type hypertension, education level only interacted with any symptomatic dizziness and dizziness problems. In general, these associations were more significant in individuals with higher education levels. Additionally, age interacted with Hcy concerning balance and falling problems, with the associations being more evident in individuals under 60 years old. Diabetes status specifically interacted with Hcy about positional dizziness, showing a significant positive correlation between Hcy and positional dizziness in diabetic patients.

### Sensitivity analysis of the association between Hcy, HHcy, and H-type hypertension and different symptomatic dizziness

We redefined HHcy and H-type hypertension using a diagnostic threshold of 10 μmol/L and conducted sensitivity analyses (Supplementary File S1). In the fully adjusted logistic regression model (Model 3), HHcy remained significantly associated with increased risks of experiencing any symptomatic dizziness (OR: 1.31, 95% CI: 1.06–1.62), balance problems (OR: 1.41, 95% CI: 1.14–1.74), and fall issues (OR: 1.80, 95% CI: 1.32–2.45). However, the association between HHcy and dizziness problems became non-significant (OR: 1.24, 95% CI: 0.98–1.56). H-type hypertension, defined using a lower threshold, maintained significant positive associations with various symptomatic dizziness in the fully adjusted model. These associations included any symptomatic dizziness (OR: 1.59, 95% CI: 1.27–1.98), dizziness problems (OR: 1.49, 95% CI: 1.18–1.88), balance problems (OR: 1.61, 95% CI: 1.28–2.03), and falling problems (OR: 1.56, 95% CI: 1.13–2.15). Consistent with the primary analysis, neither HHcy (OR: 1.04, 95% CI: 0.72–1.50) nor H-type hypertension (OR: 1.25, 95% CI: 0.78–2.00) showed significant associations with positional dizziness in the fully adjusted model. Sensitivity analyses confirmed robust positive associations between HHcy and H-type hypertension with non-positional dizziness symptoms, regardless of whether 10 or 15 μmol/L was used as the diagnostic threshold.

## Discussion

This study, based on a large-scale NHANES dataset comprising 6,970 participants, is the first to systematically investigate the associations between Hcy, HHcy, H-type hypertension, and various types of dizziness symptoms. It is noteworthy that previous studies have preliminarily revealed the potential link between HHcy and dizziness, laying the foundation for our understanding of this complex relationship. Blum’s prospective study, though limited to 68 participants (37 vitamin B12-deficient patients vs. 31 controls), demonstrated that HHcy induced by B12 deficiency could lead to abnormal vestibular evoked myogenic potentials (VEMP) (39). Additionally, Alexis Lion et al.’s study, focusing on 61 non-institutionalized elderly women, found a significant association between Hcy levels ≥12 μmol/L and dizziness, potentially through impaired vestibular-visual integration resulting in balance disorders (40). They hypothesized that elevated Hcy levels might lead to balance disorders by affecting vestibular-visual integration, providing important clues for understanding the relationship between Hcy and dizziness from a neurophysiological perspective. They hypothesized that elevated Hcy levels might lead to balance disorders by affecting vestibular-visual integration, offering valuable insights into the neurophysiological mechanisms underlying the association between Hcy and dizziness.

However, it is important to note that these earlier studies have certain limitations. Firstly, they were conducted with relatively small sample sizes. Secondly, the study populations were quite specific; for instance, Blum’s research focused on patients with vitamin B12 deficiency, while Alexis Lion’s study concentrated on older women. These factors may limit the generalizability of their conclusions. Our findings align with prior research by Blum and Alexis Lion, further supporting the association between Hcy, HHcy, and symptomatic dizziness. It is noteworthy that our study employed a relatively large sample size, which may help reduce uncertainties associated with insufficient sampling and potentially enhance the stability of results and their applicability to similar populations. Furthermore, this research comprehensively examined Hcy, HHcy, and H-type hypertension in conjunction, exploring their associations with various types of symptomatic dizziness. This multifactorial analysis may contribute to understanding the mechanisms by which HHcy influences the onset and progression of dizziness, offering new perspectives and deeper insights. Collectively, these findings extend beyond mechanistic insights to offer valuable clinical implications for both prevention strategies and management approaches in the dizziness population.

The association between Hcy, H-type hypertension, and symptomatic dizziness likely involves multi-system pathophysiological mechanisms. From a vascular function perspective, Hcy can impair blood vessel function by activating oxidative stress and inflammatory responses (41, 42). Pushpakumar et al. (43) demonstrated that Hcy stimulates reactive oxygen species (ROS) generation in endothelial cells and suppresses nitric oxide (NO) synthesis, resulting in vascular inflammation and compromised endothelial function. The vestibular system is highly sensitive to blood flow regulation, and this endothelial dysfunction can lead to local hypoperfusion, potentially triggering dizziness symptoms (44). Particularly noteworthy is the co-occurrence of hypertension and HHcy, referred to as H-type hypertension, which through their additive effects substantially impairs cerebral perfusion, thereby amplifying the susceptibility to ischemic dizziness (34, 45). Clinical studies have demonstrated that, compared to patients with hypertension alone, those with H-type hypertension exhibit a significantly increased total burden of cerebral small vessel disease (OR = 5.028, 95% CI: 2.323–10.883). Moreover, a clear synergistic effect between hypertension and HHcy on cerebral small vessel disease was observed (OR = 2.776, 95% CI: 1.564–4.927) (46). Patients with H-type hypertension have a higher incidence of cerebral microbleeds (CMBs), as well as more severe white matter hyperintensities (WMH) and perivascular space (PVS) lesions, particularly in the posterior circulation territory (47–49). These hemodynamic changes in the posterior circulation may constitute the key pathological basis for the increased susceptibility to ischemic dizziness in this patient group.

In addition to vascular mechanisms, Hcy may also directly act on vestibular neurons, causing excitotoxicity through overactivation of glutamate receptors and increased calcium influx, thereby interfering with vestibular signal transmission (50). This neurotoxic effect is consistent with our clinical observations - patients with HHcy not only present with simple dizziness but are also often accompanied by balance dysfunction. Particularly noteworthy is that our data demonstrates the most prominent association between HHcy and fall problems (OR = 1.83, 95% CI: 1.24–2.77). Although the hypothesis that HHcy affects the vestibular-spinal reflex pathway and leads to postural control abnormalities remains to be verified (51, 52), previous studies have confirmed that HHcy indeed increases the risk of falls in older adults through skeletal muscle system dysfunction (53, 54). The underlying molecular mechanisms may also involve epigenetic regulation. Recent studies suggest that Hcy as a methyl donor, participates in DNA methylation processes (55). This involvement could potentially influence vestibular function by altering the expression patterns of vestibular-related genes. While this mechanism has been confirmed in other neurological disorders (56, 57), its specific role in vestibular dysfunction requires further investigation.

Nevertheless, our study failed to find a significant association between Hcy-related factors and positional dizziness, aligning with previous findings. Rather than vascular or metabolic factors, positional vertigo primarily stems from otolith organ dysfunction, specifically otoconia dislodgement and otolithic membrane degeneration (58, 59), demonstrating distinct pathophysiological mechanisms from other dizziness types.

Our subgroup analysis further uncovered that the relationship between Hcy, HHcy, H-type hypertension, and dizziness symptoms is influenced by several factors. Sex and education level demonstrated the most notable interactions, which is consistent with the previous view of Katzenberger et al. (60) that male patients and those with higher education levels are more likely to be referred for further dizziness evaluation. Consequently, this indicates that in clinical practice, we may develop targeted screening and intervention approaches for male patients. Moreover, an interaction between age and Hcy was observed in balance and falling problems, with a stronger correlation among individuals under 60 years old. This discovery implies that we might be able to prevent dizziness in younger and middle-aged populations by lowering Hcy levels through vitamin B6 and folic acid supplementation (61, 62). This study, utilizing the large-scale NHANES dataset, provides potential epidemiological insights into the associations between Hcy, HHcy, H-type hypertension, and symptomatic dizziness. The findings suggest that close attention should be paid to Hcy levels when evaluating patients with dizziness, especially those with hypertension. However, this study has several limitations that warrant further research and improvements.

Firstly, this study relied on questionnaires to categorize dizziness types, which differs from direct clinical assessments of vestibular function. Questionnaires may introduce recall bias and subjectivity, potentially failing to accurately distinguish between vestibular, non-vestibular, or functional dizziness. Self-reported fall data, especially in older adults, is susceptible to recall bias. Older adults may forget, deny, or underreport falls due to embarrassment, potentially leading to underestimation of actual fall rates. Additionally, the NHANES balance questionnaire lacked a standardized definition of “dizziness,” allowing participants to interpret questions based on their understanding, which may result in heterogeneous symptom reporting. Secondly, the inherent nature of cross-sectional studies limits our ability to establish causal relationships between HHcy, H-type hypertension, and various types of dizziness. While our study reveals potential associations, it cannot definitively determine whether HHcy or H-type hypertension are direct causes of dizziness or merely accompanying factors. Therefore, future research must overcome the limitations of cross-sectional studies by conducting rigorous prospective cohort studies to elucidate the potential causal links between Hcy, HHcy, H-type hypertension, and different types of symptomatic dizziness.

To address the limitations of questionnaire-based diagnoses, future studies should adopt the International Classification of Vestibular Disorders (ICVD) criteria for more precise dizziness classification. Additionally, to comprehensively assess vestibular function, research should incorporate standardized clinical vestibular assessment tools such as the HINTS examination (63, 64) and Dix-Hallpike test (65), along with objective vestibular function tests. These include the video head impulse test (vHIT) (66) for semicircular canal function, VEMP (67) for otolith and vestibular pathway function, and rotary chair testing (68, 69) for comprehensive vestibulo-ocular reflex evaluation. This integrated research approach will enable future studies to more accurately differentiate dizziness subtypes and explore potential correlations between HHcy /H-type hypertension and specific vestibular pathway dysfunctions. Moreover, it will facilitate the investigation of links between these factors and objectively measured balance impairments and falling risks.

Furthermore, it is imperative to delve deeper into the underlying mechanisms. Future investigations should aim to elucidate the specific associations between Hcy or H-type hypertension and microperfusion in the posterior circulation and vestibular organs. The integration of advanced neuroimaging techniques, such as transcranial Doppler (TCD) (70) and arterial spin labeling MRI (ASL-MRI) (71), would facilitate precise quantitative analysis of these relationships. Concurrently, it is crucial to explore the potential direct neurotoxicity of Hcy (50), examining its effects on vestibular neuron excitability and synaptic transmission through animal models or *in vitro* experiments. Additionally, investigation of epigenetic regulatory mechanisms, particularly the analysis of Hcy-related DNA methylation changes and their impact on vestibular-related gene expression (55), warrants attention.

Lastly, we recommend conducting randomized controlled trials, when feasible, to evaluate whether strategies aimed at lowering Hcy levels through folic acid and vitamin B6/B12 supplementation can effectively prevent or alleviate specific types of dizziness symptoms, improve balance function, and reduce falling risks (61, 62). Regarding of the potential interactions of age, gender, and education level observed in our subgroup analyses, future intervention studies should also focus on these factors to explore possible specific beneficiary populations and consequently provide evidence for developing individualized screening and intervention strategies.

### Strengths and limitations

This study is the first to explore the potential associations between Hcy levels, H-type hypertension, and symptomatic dizziness. The major strength lies in its utilization of the NHANES database, which encompasses a large-scale, nationally representative sample of non-institutionalized populations, substantially enhancing the statistical power of our analysis. However, it should be noted that the data used in this study was collected between 1999 and 2004, which presents certain temporal limitations. To validate the generalizability of our findings, future studies should incorporate more recent and temporally relevant prospective cohort data for verification analyses. The cross-sectional design inherently restricts causal relationships, while self-reported symptoms are subject to recall bias and interpretational variability. Additionally, the absence of specific vestibular function tests constrains our ability to isolate vestibular effects. Despite rigorous control measures, the influence of unmeasured confounding factors cannot be ruled out. These limitations underscore the necessity for future longitudinal and interventional studies to validate and extend our findings.

## Conclusion

This study revealed that Hcy levels, HHcy, and H-type hypertension were significantly associated with various symptomatic dizziness. Among them, HHcy was most strongly associated with falling problems, and H-type hypertension was most prominently associated with any symptomatic dizziness, while the three were not significantly associated with positional dizziness. Consequently, the recognition and control of HHcy and H-type hypertension play a vital role in dizziness management and diagnosis.

## Data Availability

The original contributions presented in the study are included in the article/Supplementary material, further inquiries can be directed to the corresponding author.

## Footnotes

^1^ https://www.cdc.gov/nchs/nhanes/irba98.htm
