# Supplementary material for: Homocysteine, HHcy, H-type hypertension and dizziness: an NHANES analysis
Source: Front Neurol. 2025 Jul 14;16:1550568. doi: 10.3389/fneur.2025.1550568 (PMC12301206; doi:10.3389/fneur.2025.1550568)
Supplement: Supplementary file 1 [file Table_1.docx]

**S1：Association between Hcy, HHcy, and H-type hypertension and different symptomatic dizziness**

|  | | Any.Symptomatic.dizziness | | | Dizziness.problems | | | Balance.problems | | | Falling.problems | | | Positional.dizziness | | |
| --- | --- | --- | --- | --- | --- | --- | --- | --- | --- | --- | --- | --- | --- | --- | --- | --- |
| **Model** | **Characteristic** | **OR** | **95% CI** | **p-value** | **OR** | **95% CI** | **p-value** | **OR** | **95% CI** | **p-value** | **OR** | **95% CI** | **p-value** | **OR** | **95% CI** | **p-value** |
| Model 1 | HHcy |  |  |  |  |  |  |  |  |  |  |  |  |  |  |  |
|  | No | — | — |  | — | — |  | — | — |  | — | — |  | — | — |  |
|  | Yes | 1.604 | 1.362, 1.888 | **<0.001** | 1.401 | 1.179, 1.665 | **<0.001** | 1.887 | 1.599, 2.228 | **<0.001** | 2.710 | 2.087, 3.520 | **<0.001** | 1.132 | 0.8338, 1.538 | 0.4 |
|  | H.hypertension |  |  |  |  |  |  |  |  |  |  |  |  |  |  |  |
|  | No | — | — |  | — | — |  | — | — |  | — | — |  | — | — |  |
|  | Yes | 2.159 | 1.859, 2.508 | **<0.001** | 1.787 | 1.533, 2.083 | **<0.001** | 2.412 | 1.987, 2.929 | **<0.001** | 2.882 | 2.201, 3.774 | **<0.001** | 1.496 | 1.033, 2.166 | **0.034** |
| Model 2 | HHcy |  |  |  |  |  |  |  |  |  |  |  |  |  |  |  |
|  | No | — | — |  | — | — |  | — | — |  | — | — |  | — | — |  |
|  | Yes | 1.505 | 1.240, 1.827 | **<0.001** | 1.423 | 1.151, 1.759 | **0.002** | 1.643 | 1.343, 2.011 | **<0.001** | 2.271 | 1.690, 3.050 | **<0.001** | 1.236 | 0.8771, 1.741 | 0.2 |
|  | H.hypertension |  |  |  |  |  |  |  |  |  |  |  |  |  |  |  |
|  | No | — | — |  | — | — |  | — | — |  | — | — |  | — | — |  |
|  | Yes | 1.816 | 1.503, 2.195 | **<0.001** | 1.672 | 1.363, 2.051 | **<0.001** | 1.880 | 1.522, 2.323 | **<0.001** | 2.032 | 1.507, 2.740 | **<0.001** | 1.483 | 0.9693, 2.269 | 0.068 |
| Model 3 | HHcy |  |  |  |  |  |  |  |  |  |  |  |  |  |  |  |
|  | No | — | — |  | — | — |  | — | — |  | — | — |  | — | — |  |
|  | Yes | 1.309 | 1.057, 1.622 | **0.015** | 1.238 | 0.9823, 1.561 | 0.069 | 1.407 | 1.138, 1.738 | **0.003** | 1.796 | 1.316, 2.452 | **<0.001** | 1.039 | 0.7184, 1.502 | 0.8 |
|  | H.hypertension |  |  |  |  |  |  |  |  |  |  |  |  |  |  |  |
|  | No | — | — |  | — | — |  | — | — |  | — | — |  | — | — |  |
|  | Yes | 1.589 | 1.273, 1.982 | **<0.001** | 1.488 | 1.177, 1.880 | **0.002** | 1.613 | 1.279, 2.034 | **<0.001** | 1.561 | 1.134, 2.150 | **0.008** | 1.246 | 0.7769, 1.999 | 0.3 |
| Abbreviations: CI = Confidence Interval, OR = Odds Ratio | | | | | | | | | | | | | | | | |

HHcy: using a diagnostic threshold of 10 µmol/L
